# Protective Factors That Foster Resilience to HIV/AIDS: Insights and Lived Experiences of Older Gay, Bisexual, and Other Men Who Have Sex with Men

**DOI:** 10.3390/ijerph18168548

**Published:** 2021-08-13

**Authors:** Renato M. Liboro, Tammy C. Yates, Sherry Bell, Brandon Ranuschio, George Da Silva, Charles Fehr, Francisco Ibañez-Carrasco, Paul A. Shuper

**Affiliations:** 1 Department of Psychology, University of Nevada, Las Vegas, NV 89154, USA; bells12@unlv.nevada.edu (S.B.); fraga@unlv.nevada.edu (B.R.); 2 Centre for Addiction and Mental Health, Institute for Mental Health Policy Research, Toronto, ON M5S 2S1, Canada; george.dasilva@camh.ca (G.D.S.); charles.fehr@camh.ca (C.F.); paul.shuper@camh.ca (P.A.S.); 3 *Realize*, Toronto, ON M5R 2A7, Canada; tyates@hivandrehab.ca; 4 Dalla Lana School of Public Health, University of Toronto, Toronto, ON M5T 3M7, Canada; francisco.ibanez.carrasco@utoronto.ca

**Keywords:** protective factors, interventions, resilience to HIV/AIDS, gay, bisexual, other men who have sex with men

## Abstract

Since the beginning of the HIV/AIDS epidemic, gay, bisexual, and other men who have sex with men (gbMSM) have been disproportionately impacted by HIV/AIDS health disparities. Research showed that resilience to HIV/AIDS is associated with increased use of relevant health services, lower sexual health risks, and improved mental health outcomes among racially and ethnically diverse gbMSM. As the subpopulation that has historically been impacted by HIV/AIDS the longest, older gbMSM living with HIV/AIDS have inarguably exhibited resilience to HIV/AIDS the most. The qualitative study described in this paper sought to identify and examine protective factors that fostered resilience to HIV/AIDS based on the insights and lived experiences of racially and ethnically diverse, older gbMSM. Applying a community-based participatory research approach that included the meaningful involvement of older gbMSM living with HIV/AIDS in different roles (i.e., advisory committee member, collaborator, peer researcher, and participant), the study recruited and included forty-one older gbMSM living with HIV/AIDS from Ontario, Canada, in confidential, semi-structured interviews. Utilizing thematic analysis, we identified three major themes from the participant interviews as factors that fostered the resilience of older gbMSM to HIV/AIDS and helped to address HIV/AIDS health disparities: (1) established protective factors, (2) behavioral protective factors, and (3) controversial protective factors. This paper argues for the importance of valuing and capitalizing on these protective factors in the conceptualization and development of interventions, services, and programs that are dedicated to fostering resilience to HIV/AIDS.

## 1. Introduction

Since the beginning of the HIV/AIDS epidemic over forty years ago, gay, bisexual, and other men who have sex with men (gbMSM) have been disproportionately affected by HIV/AIDS health disparities, especially in Canada and the United States [1,2,3,4,5,6,7]. Moreover, research has shown that over the years, the HIV/AIDS epidemic has become inextricably linked to other forms of health disparities and social problems (i.e., psychological comorbidities, problematic substance use, sexual victimization, stigmatization, and different forms of discrimination) that have also disproportionately affected gbMSM [6,8]. In recent years, despite continuing to account for only a small fraction of the male population of North America [7,9], gbMSM have remained the subpopulation that is most adversely affected by the clinical and social impacts of HIV/AIDS, representing approximately 52% of new HIV cases in Canada [3,7] and nearly 69% of new HIV cases in the United States [4] by the end of 2018. According to recent statistical reports, the vast majority of newly diagnosed cases of HIV/AIDS have involved racial and ethnic minority gbMSM [3,4,6], who appear to experience persistent access barriers to HIV-related healthcare and services, and have lower levels of awareness and knowledge of biomedical interventions to mitigate the risks of HIV transmission [8,10,11].

Despite the documented elevated levels of poor general health, physical limitations, and mental distress they have experienced compared to their heterosexual counterparts, racially and ethnically diverse, older gbMSM and other sexual and gender minorities living with HIV/AIDS have displayed remarkable resilience (i.e., the ability to successfully handle or surmount adversity and challenges) for decades, aging successfully with strong personal and social ties to the community [12,13]. Researchers focused on strengths-based approaches for developing interventions to foster resilience to HIV/AIDS have not only scrutinized the different pathways involved in fostering the resilience of older gbMSM to HIV/AIDS [14] but have also recognized the role of resilience as a critical facilitator of the increased use of HIV-related health services among gbMSM living with HIV/AIDS, as well as the lower sexual health risks, improved HIV care, and physical and mental health outcomes that are associated with such increased use of HIV-related health services [15,16].

Recent empirical studies have been conducted to help identify and explore unique opportunities to foster resilience to HIV/AIDS by building strong “villages” (i.e., social networks), reducing structural inequities, and enhancing HIV treatment and care systems in collaboration with community stakeholders [17], including opportunities that were sustained with the support of different AIDS service organizations (ASOs) and other community-based agencies [18,19,20,21,22]. ASOs have been actively involved in studies investigating broad multisectoral interventions that address income, housing, substance use, and mental health issues [18]; underscore the crucial role of non-medical, community-based workers in providing services to gbMSM in the HIV care continuum [20]; and highlight the peer support, community leadership, advocacy, and active participation of gbMSM, which have all been central to the HIV response since the epidemic began [19,22].

It was the aim of our study to examine protective factors that fostered resilience to HIV/AIDS based distinctly on the unique insights and lived experiences of racially and ethnically diverse, older gbMSM living with HIV/AIDS, who have historically exhibited remarkable resilience to HIV/AIDS in North America since the start of the epidemic [12,13,14]. Such protective factors may prove extremely edifying and useful in the conceptualization and development of interventions that are to be implemented by ASOs and other community-based agencies and groups that are dedicated to fostering the resilience of gbMSM to HIV/AIDS and addressing HIV/AIDS health disparities in the 21st century.

For the purpose of guiding our study, we defined and conceptualized resilience to HIV/AIDS as the capacity of gbMSM to: (1) survive the clinical and social impacts of HIV/AIDS; (2) live full lives despite a chronic illness; (3) thrive despite the challenges brought about by HIV stigma and discrimination; and/or (4) meaningfully contribute to the goal of ending the HIV/AIDS epidemic.

## 2. Materials and Methods

### 2.1. Partnerships and Collaborations

Our research team conducted a qualitative study that utilized a community-based participatory research approach [23,24] in collaboration with numerous community partners from across Central and Southwestern Ontario, Canada. Our primary community partner, *Realize* (https://www.realizecanada.org/en, accessed on 12 August 2021), was instrumental in the creation of our community advisory board (CAB), which was composed of various relevant stakeholders, including older gbMSM, who provided invaluable input on all aspects of our research process. Most of our community partners were ASOs and community-based LGBTQ+ agencies that were keen to learn more about protective factors that could prospectively help to foster resilience to HIV/AIDS among gbMSM and develop interventions that would address HIV/AIDS health disparities. Our team also collaborated directly with older gbMSM, who became meaningfully involved in our study not only as CAB members, participant recruiters, and study participants but also as peer researchers with active engagement in every step of our year-long study. Our team and community partners worked together to create our study protocol, which was approved by the Research Ethics Board (REB) of the Centre for Addiction and Mental Health (CAMH) in Toronto, Ontario, Canada.

### 2.2. Participants and Procedures

The results of our study discussed in this paper were from part of a larger study that focused on identifying and investigating multiple aspects of resilience to HIV/AIDS among older gbMSM. Among all the participants recruited for the larger study, 41 racially and ethnically diverse, older gbMSM living with HIV/AIDS took part in the confidential, audio-recorded, semi-structured interviews of the study described in this paper. Participants were eligible to join our study if they were: (1) willing to disclose their HIV status for the purposes of the study; (2) males who identified as gay, bisexual, or men who have sex with men; (3) 40 years of age or older; and (4) residing in Ontario, Canada, at the time of the study. We had a diverse participant population in terms of race and ethnicity, age range, how participants identified with regard to their sexuality, and geographical location (see Table 1).

We obtained informed consent from our participants prior to the start of their interviews. We utilized an interview guide that was pre-approved by the CAMH REB. Our interview guide was composed of open-ended questions that were designed to obtain the insights of our participants on protective factors that they believed fostered resilience to HIV/AIDS. Our participants were co-interviewed by the first author and one of the study’s two peer researchers. We continued to recruit and interview participants until we were no longer able to derive any new information based on our growing data set (i.e., point of data saturation). We assigned each participant a pseudonym and study number from the time of their interview and compensated each participant with 25 CAD for their time and efforts to support our study. Each interview was transcribed verbatim by the peer researcher who took part in the interview, and each of the transcripts was cross-checked by the first author for further verification.

### 2.3. Analysis of Data

The first author and two peer researchers who conducted the interviews comprised the panel of coders who conducted the initial step for the thematic analysis [25] of the interview data. As individual coders, the first author and peer researchers reviewed and coded ten representative transcripts for major themes, sub-themes, and representative quotes. They then met to compare themes, sub-themes, and quotes in order to finalize a thematic codebook, which the first author used to code and analyze the remaining 31 transcripts for the creation of a comprehensive, community report with aggregated and de-identified data. Next, the rest of the team and the CAB subsequently reviewed and provided their feedback on the community report. The community report was later revised to incorporate the feedback provided by the team and the CAB and then shared with our community partners and collaborators for distribution and knowledge mobilization.

## 3. Results

We were able to identify three major themes from the analysis of our interview data: (1) established protective factors, (2) behavioral protective factors, and (3) controversial protective factors, all of which our participants believed fostered their resilience to HIV/AIDS. Each major theme included sub-themes (i.e., specific protective factors) that were described by participants in the interviews.

### 3.1. Established Protective Factors

During the interviews, participants discussed factors that were fairly established in prior academic literature as factors that afford a certain level of protection to gbMSM from the clinical and social impacts of HIV/AIDS. These factors included sub-themes such as *education*, *religion and spirituality*, and *social support from family and friends*.

According to participants (*n* = 10; 24%), HIV *education* (i.e., acquiring information or knowledge on HIV from training or study) was a significant influence on how they made important decisions regarding their physical and mental health, as well as the kind of care they accessed and received from community-based health and social services. They felt that the more information they gathered and understood, the better decisions they made when it came to accessing HIV care and attaining desirable health outcomes. In particular, education on how to prevent HIV transmission, how to remain healthy in terms of suppressing their viral load and maintaining their CD4 counts, and the latest information on HIV/AIDS were the most salient to participants. Peter, who had been residing in Toronto and living with HIV/AIDS for over a decade, explained, “Well, knowledge is power. If people are able to get the information and knowledge they need from HIV education, they would be able to manage their health more easily and have more peace of mind.” Participants claimed that they gained education about matters that were most relevant to them from different sources, including information shared by their healthcare and service providers, information pamphlets from clinics, community reports from community-engaged researchers, lunch and learns, and other HIV education events sponsored by ASOs and highly recommended and reliable online resources.

Participants also discussed the importance of *religion and spirituality* in fostering their resilience to HIV/AIDS. Although many participants revealed that religion, its tenets, and practices had made their lives difficult growing up, they also shared how much they valued the spirituality they had that was rooted in their religious upbringing and background. They felt that their spirituality was a crucial factor that protected them from the clinical, and more so, the social impacts of living with HIV/AIDS, such as stigma and discrimination. Several participants turned to their religion later in life, receiving comfort from knowing that they could believe in a higher power and a community that will accept them unconditionally. Gerry, who was born in the Greater Toronto Area and raised Catholic, expressed, “I am still a Christian and a person of faith. I go to church and pray regularly. This keeps me grounded and strong, especially during really tough times.” Mandy, who was diagnosed with HIV shortly after immigrating from China to Canada, shared, “I don’t attend church because people there eventually ask what I do. I do sex work to survive. But I keep my faith. I know God loves me and that He understands.” While some participants were still strongly tied to their religion, others felt they stayed just as resilient by remaining spiritual without organized religion.

A large majority of participants (*n* = 24; 59%) attributed their resilience to HIV/AIDS to their *social support from family and friends*. Most of these participants (*n* = 18; 75%) were eager to admit that not everyone in their family and close circle of friends were able to provide the unconditional support they needed, especially during their most challenging times. However, many participants explained that often, even if only one or two family members or friends stuck by them after they disclosed their HIV diagnosis, the support and loyalty they received was enough to help them through their difficult days. A devoted mother, a reliable brother, or an old and dear childhood friend, was all they needed to muster the fortitude to keep going during their prolonged hospitalizations and recovery. Joey, who lived in the Greater Toronto Area and identified as bisexual, recounted how difficult it was for his sister after he was diagnosed with HIV, “My sister was okay about it after a while. She could not wrap her head around it at first but it was never an issue with her children. She eventually came around with her kids’ help and became very supportive.” Other participants were not as fortunate to have supportive family members but had numerous gbMSM friends who provided a strong support system. Chris, who grew up in Downtown Toronto, narrated, “Four of us lived in one house in the 90′s. It was rough, but what happened in that house, stayed in that house…we supported each other no matter what. We were chosen family and remain friends to this day.”

### 3.2. Behavioral Protective Factors

Participants described how they implemented particular behavioral strategies that afforded them some layer of protection from the clinical and social impacts of HIV/AIDS over the years. These behavioral strategies were described in prior academic literature, but as far as we could determine, were minimally discussed in the context of fostering resilience to HIV/AIDS among racially and ethnically diverse, HIV-positive, older gbMSM. These behavioral protective factors represented sub-themes, such as *compartmentalizing*, *serosorting*, and *volunteering*.

Many participants (*n* = 31; 76%) revealed the enormous challenges they experienced having to deal with sexual minority discrimination and HIV stigma in several aspects of their lives. In order to avoid embarrassment, judgment, or rejection, participants realized early on during their struggles that *compartmentalizing* certain aspects of their lives, particularly by keeping their sexuality and HIV status separate or concealed from their work and families, just made their lives easier, at least until their circumstances would get better or they were able to find more suitable solutions to their challenges. For the most part, participants who chose to compartmentalize decided that they would only be open about their sexuality and their HIV status to other gbMSM, especially those whom were either their friends or sexual partners. Mpenda, who was already HIV-positive before migrating to Canada as a refugee, expounded on his conscious strategy to avoid more difficulties and foster his resilience, “They still have their biases. If you meet me in my [local] Kenyan community, I am very careful with others even if they are refugees themselves. I don’t share my HIV status…and I tread with a lot of caution.” Benny echoed similar sentiments as he described how he survived and adjusted in the Greater Toronto Area during the early years after his HIV diagnosis, “As an Asian man who is gay and HIV-positive, I kept three things separate. Work, home, and my gay life…they were compartmentalized for nearly seven years. Back then, that’s how I coped. I regularly avoided the Asian community.” Although they recognized that compartmentalizing did not necessarily directly contribute to fostering their resilience, participants believed that this behavioral strategy provided them reprieve that allowed them to foster their resilience in other more direct ways.

Another behavioral strategy that our participants implemented in order to mitigate the clinical and social impacts of HIV/AIDS was *serosorting*. By choosing to engage in sexual activity only with other HIV-positive gbMSM, participants believed they were not only able to increase the convenience of selecting prospective sexual partners, but they were also able to decrease significant sources of stressors, such as navigating status disclosure issues, embarrassment, and most importantly, rejection. According to participants, serosorting was not difficult to do because of the availability of online and mobile phone app dating options that either gave them the opportunity to identify themselves upfront as HIV-positive on their profile or allowed them to meet others in a space that was exclusively for HIV-positive individuals. These options saved them time and from the trouble of having to sort through hundreds of dating profiles, as well as navigating through the challenges of disclosing their HIV status and responding to unfavorable reactions to their disclosure. Robert, who sought out other gbMSM in Downtown Toronto solely through mobile phone dating apps, related, “I always looked to hook up with people who were HIV-positive. I didn’t want to deal with having to disclose later on…or the possible rejection. Things were better this way. Both parties are already aware of each other’s status beforehand.” Victor felt almost the same way, especially about having to face rejection, “I am extremely shy and I want to avoid any possible confrontation. I don’t know if my self-esteem is too low, but the idea of having to repeatedly experience rejection…it’s just too much for me.” Similar to compartmentalizing, serosorting did not seem to directly foster the resilience of gbMSM to HIV/AIDS, but it also offered a level of protection to our participants from the distressing social impacts of HIV/AIDS. As a protective factor, serosorting prevented the erosion of the resilience to HIV/AIDS our participants had already amassed, thereby indirectly allowing their resilience to foster and continue to grow.

Finally, our participants also relied on *volunteering* as a behavioral protective factor that they believed helped to foster their HIV resilience. Unlike compartmentalizing and serosorting, volunteering was a behavioral strategy that clearly had protective effects that indirectly and directly fostered resilience to HIV/AIDS based on the lived experiences of our participants. The vast majority of the racially and ethnically diverse, older gbMSM living with HIV/AIDS who joined our study had purposely volunteered their time and efforts to work at LGBTQ+ not-for-profit agencies and/or ASOs at some point since their HIV diagnosis. Participants asserted that volunteering provided them with several advantages that they had no doubt afforded them both protection from the adverse effects of HIV/AIDS and increased their capacity to foster their resilience. By volunteering, they were able to gain abundant opportunities to mentor others whom they felt could benefit from their knowledge and lived experiences, a feeling of being able to give back or pay it forward to their community, and a greater sense of productivity. Volunteering also provided them with many occasions to socialize and build their networks, learn new information about HIV science and treatments, and gain easier access to services and programs at community-based organizations that were relevant to them. When we asked him why it was important for him to volunteer, Mike, who had been living and availing of HIV services in rural Southwestern Ontario for over five years, replied, “It feels good to be able to help people. Sometimes it’s difficult not to judge other people’s choices when you meet them. But when you’re volunteering, you don’t do that. You’re there to help, and knowing that feels good.” Other participants had related responses to the same query. David from Downtown Toronto answered, “I get opportunities to participate in advocacy work, even activism. It’s more than just stuffing condoms. It makes me fulfilled because I believe the work is important. It also gives me easier access to programs I like [at ASOs].” Bill, on the other hand, had a distinctive story, “Volunteering was absolutely helpful to me! It was part of my overall recovery. It allowed me to build confidence and learn how to survive.” He then added, “Soon after my diagnosis, my entire life revolved around volunteering those three hours. It gave me purpose. If I could get out of bed, hold it together for three hours, it was a good week.”

### 3.3. Controversial Protective Factors

There were some factors (i.e., sub-themes) that kept reoccurring in the conversations we had during our interviews. Although these factors were the topics of previous empirical studies that focused on the mental health and wellbeing of HIV-positive gbMSM, they were not gleaned and examined directly from the insights and lived experiences of racially and ethnically diverse, older gbMSM living with HIV/AIDS in the aforementioned prior research, specifically for the purposes of discussing factors that foster resilience to HIV/AIDS. Apart from having been discussed repeatedly by many of our participants, the main commonality of these factors rested on the understanding that, although some scholars may find them controversial because they inspire much debate in extant research discourse, our participants believed that these factors not only afforded them protective effects against the clinical and social impacts of HIV/AIDS over the years, but these factors also fostered their resilience to HIV/AIDS based on their lived experiences. These factors include *abstinence*, *trauma from the loss of many lives to HIV/AIDS at the start of the epidemic*, “*managed*” *substance use*, and *meaningful sexual relationships*.

Several of our participants (*n* = 11; 27%) revealed that as younger adults, they were once married to women, had traditional nuclear families with biological children, and tried to customarily lead their lives as heterosexual men. Most of these participants were raised in religious and conservative families, felt they grew up with sheltered lives, and lived in places that provided them with very little opportunities to learn about same-sex attraction, explore their sexual orientation and non-heterosexual urges, and engage in sexual activities as men who desired to have sex with other men. In other words, their circumstances in the earliest decades or so of their lives practically compelled them to exist in a world of enforced heteronormativity and they hardly made any efforts to live to the contrary. Even as they became fully aware of their own sexual urges that were different from most other men’s, and even after having had chances to experience occasional dalliances with other men, they still chose (for the most part) to voluntarily deny themselves the opportunity to embrace their true sexuality, and instead, practiced *abstinence* from same-sex activities. In their interviews, these participants also revealed that they believed these prolonged periods of abstinence, particularly at the beginning and at the peak of the epidemic, afforded them protection from the risks and clinical and social impacts of HIV/AIDS. Ethan, who identified as bisexual and lived in the Greater Toronto Area, narrated, “I was already in my third year in college when I first went to a gay bar. I met somebody and it wasn’t a great experience. I felt guilty about it. I ended up getting married and had two kids.” Ethan further explained, “I didn’t have a lot of experience. My repertoire wasn’t large as far as gay activities. In fact, I didn’t become HIV-positive till 2013, and I know it was because I was doing things so infrequently before then.” For certain scholars, abstinence remains controversial as a strategy to be included in the development of interventions for HIV care. However, from the insights of several participants of our study, it was a factor that afforded them protective effects, in terms of both HIV acquisition, as well as transmission after being diagnosed with HIV.

Participants knew that practicing abstinence would help protect them from acquiring various antibiotic-resistant sexually transmitted infections, which are more challenging to manage medically among gbMSM living with HIV/AIDS, who may or may not be immunocompromised. For some of our participants who had been living with HIV/AIDS for many years, fostering their HIV resilience not only meant making the effort to protect themselves from the clinical and social impacts of HIV/AIDS but also trying to make a difference in terms of their advocacy and activism, particularly by doing their part in contributing to the goal of ending the HIV/AIDS epidemic. From the lessons they learned on how their own abstinence could potentially prevent the transmission of HIV to other gbMSM, practicing more self-restraint, or at least being more judicious about their choices regarding the frequency of their sexual activities, was something they believed was worth considering in order to foster their wellbeing and their resilience to HIV/AIDS.

During the first two decades of the HIV/AIDS epidemic, there was another phenomenon that swept North America, which deeply affected many sexually active gbMSM. This was the considerable *trauma that resulted from the significant loss of lives of gbMSM to HIV/AIDS*. Innumerable partners, lovers, sons, brothers, friends, peers, and familiar faces were lost to HIV/AIDS, and many gbMSM who experienced the trauma from this devastating, massive loss were overwhelmed with grief and stricken with fear. According to our participants, the grief and fear many experienced led to depression, hopelessness, isolation, extreme caution, survivor’s guilt, intense paranoia, and celibacy. Terry, who had been living with HIV/AIDS since the 1990s, shared, “People were dropping like flies…I was going to roughly two funerals a week. I was burying one very good friend after another. Many of us who were still alive either got extremely paranoid or stopped having sex altogether.” Apparently, it was not until effective combination antiretroviral therapy medications with greater safety profiles and more tolerable side effects became available that many surviving HIV-positive gbMSM started to experience a modicum of relief from the trauma of losing so many people they knew and cared for to HIV/AIDS. Although others may find the idea that there could have been some good that resulted from such trauma either ludicrous or even offensive, some participants pointed out that their experience turned out to be paradoxically protective, and later, resilience fostering. Similar to the practice of abstinence, the trauma of losing so many lives of gbMSM to HIV/AIDS made some participants consider the recognition of such traumatic loss as a protective factor that could be utilized as a strong motivation for fiercely working toward the goal of ending the HIV/AIDS epidemic, and thus, an impetus for fostering their HIV resilience.

One of the more polemical factors that participants discussed as they recalled their lived experiences of struggling through many challenges due to HIV/AIDS was what they considered *“managed” substance use*. At least a quarter of our participants claimed that their use of illicit drugs was a coping mechanism that definitely helped them get through harrowing experiences, such as family rejection, job loss, isolation, depression, housing and income insecurity, stigma, discrimination, and condemnation. For many, they felt they were able to “manage” their substance use because they only used marijuana, mushrooms, and ecstasy to cope, but no cocaine, methamphetamines, or heroin. Others believed that their substance use was “managed” because they never did intravenous drugs. Still others believed that as long as their substance use was with other people they knew and trusted, and only on occasions when their environment and setting were within their own control and choice, they considered their substance use to be “managed.” For a few though, many years of using stronger, more dangerous substances was necessary to get them through the darkest, most difficult days of their lives. Several participants reported that there were many years of their lives lost to substance use. Dan, who lived in Downtown Toronto and was very familiar with its street-based drug scene, shared, “Sounds odd but without drugs all those years, I may not have lasted this long. I was messed up, but doing drugs was all I could do to make it through the day.” At the time of their interviews, several (*n* = 8; 20%) participants were already sober or had been substance-free for years. While these participants acknowledged that they now recognize that there could have been other ways to cope with their difficult experiences living with HIV/AIDS in the earlier years after their diagnosis, they insisted that at that point in their lives, using drugs was their best or only available recourse to cope.

At the time of their interviews, almost half of our participants revealed that some of the most *meaningful relationships* they previously or currently had that helped them foster their resilience to the impacts of HIV/AIDS started out as *sexual* relationships. Some of these relationships remained sexual and monogamous in the traditional sense, some remained sexual and monogamous with a modified definition of “monogamy” (i.e., a commitment to each other in all respects except for their sexual activities), and some remained very emotionally close, platonic, and/or pragmatic, but were no longer sexual. The aspects of all these relationships that remained consistent across the board included friendship, understanding, companionship, communication, emotional connection, trust, loyalty, consistency regarding taking care of one another, sharing resources, and/or sharing a life together. In whichever way our participants defined the boundaries of their meaningful relationships (in terms of bonding, commitment, or monogamy), one thing for certain was that these relationships were not only protective in terms of withstanding the impacts of HIV/AIDS but were also bases of strong foundations for them when it came to fostering their HIV resilience. Mario, who had been in a long-term relationship with his partner for nearly 30 years, expressed, “Being in a healthy relationship is good for my sexual, mental, and overall health. If my relationship is healthy, I stay healthy.” Steven described the features of his relationship that made it a dependable source of protection and resilience: “We take care of each other. We may not be [sexually] exclusive, but our companionship, loyalty, trust, and dedication to caring for each other all go a very long way.”

## 4. Discussion

Three major themes that helped to foster our participants’ resilience to HIV/AIDS were identified: (1) established protective factors; (2) behavioral protective factors; and (3) controversial factors that our participants believed provided them protective effects against, and fostered their resilience to, HIV/AIDS. It was the aim of our study to examine protective factors that fostered resilience to HIV/AIDS based distinctly on the unique insights and lived experiences of racially and ethnically diverse, older gbMSM living with HIV/AIDS, particularly factors that may prove edifying and useful in the conceptualization and development of interventions to be implemented by ASOs and other community-based agencies that are dedicated to fostering the resilience of gbMSM to HIV/AIDS and addressing HIV/AIDS health disparities. In the sections that follow, we discuss the merits of considering, valuing, and possibly capitalizing on these protective factors in the conceptualization and development of interventions fostering resilience to HIV/AIDS, especially because they were harnessed from the insights and personal experiences of gbMSM who have in all greatest likelihood exhibited the most HIV resilience since the start of the epidemic in North America.

### 4.1. Considering and Valuing Established Protective Factors in the Development of Interventions That Foster Resilience to HIV/AIDS

Existing academic literature has reasonably established over the years that factors such as *education*, *religion and spirituality*, and *social support from family and friends* afford protective effects to gbMSM against the clinical and social impacts of HIV/AIDS, and consequently, foster their HIV resilience [16,26,27,28]. Researchers have found that HIV *education* is a major factor that protects gbMSM from the impacts of HIV/AIDS by buffering the effects of elevated levels of sexual risk behaviors that are related to HIV infections [28]. Researchers have underscored the value of educational resources in creating development opportunities, particularly opportunities to educate older people living with HIV/AIDS through community-based organizations that are seeking to expand patient or client education on HIV/AIDS issues [29]. Additionally, education was noted to have an important role in facilitating the use of health services among aging gbMSM living with HIV/AIDS, which in turn, has led to enhancing their positive physical and mental health outcomes [16]. In the last decade, ASOs, LGBTQ+ agencies, and community-based groups have already recognized the value of education as a protective factor that has vast potential for fostering HIV resilience, especially among their gbMSM clients. Many of these organizations and groups have long provided education opportunities within their priority communities, which include gbMSM, to encourage discussions about HIV, sexually transmitted infections, sexual health, and harm reduction. These opportunities have included education curricula, mentorships, resources, seminars, toolkits, and other programs on a wide array of topics that are relevant to their clients [16,29].

Much attention has focused on *religion and spirituality* as protective factors that foster resilience to HIV/AIDS among HIV-positive gbMSM. Researchers not only examined the perceived benefits of religious and spiritual coping as critical personal and social resources for older adults living with HIV/AIDS and an aging epidemic [30] but they also documented the importance of faith, spirituality, and practicing a religion as an important consideration for developing interventions that foster resilience to HIV/AIDS [26,27,31,32]. ASOs, LGBTQ+ agencies, and community-based groups have valued religion and spirituality as factors that offer protection against, and promote resilience to, the clinical and social impacts of HIV/AIDS. These organizations and groups showed recognition for the value of spirituality, religiousness, and the choice to practice religious customs by supporting researchers in the recruitment of gbMSM participants for studies that explore the importance of religion and spirituality in informing the development of interventions that would foster resilience to HIV/AIDS [30,32].

*Social support from family and friends* has also long been established by empirical studies as a protective factor that helps to foster resilience to HIV/AIDS among HIV-positive gbMSM [16,27,29,33,34]. Researchers who explored the interactions between social support from family and friends, coping, and resilience determined that social support is key to facilitating the use of health services among HIV-positive older gbMSM, as well as resisting the adverse impacts of overwhelming struggles, such as problematic substance use and homelessness [16,27,29,34]. As a recognition of the value of maximizing and capitalizing on social support from family and friends as a factor that fosters resilience among gbMSM and other people living with HIV/AIDS, ASOs and other agencies have initiated and sustained family and peer support programs over the years [13,29,34].

### 4.2. Considering and Valuing Behavioral Protective Factors in the Development of Interventions That Foster Resilience to HIV/AIDS

Although they were examined in prior academic research on HIV/AIDS, the behavioral protective factors that were described by our participants have not been (to the best of our knowledge) discussed specifically in the context of fostering resilience to HIV/AIDS. These behavioral protective factors included *compartmentalizing*, *serosorting*, and *volunteering*. Many researchers have discussed in the past decade the protective, albeit likely transient, effects that *compartmentalizing* has afforded to gbMSM who chose to categorically keep their sexual identities, encounters, activities, and HIV statuses separate from their religious identities, families, and work lives [32,35,36,37,38,39,40]. In these discussions, compartmentalizing was not considered as a behavioral protective factor that could potentially foster the resilience of gbMSM to HIV/AIDS, and more so, as a factor that could be valued and utilized in the development of interventions that are dedicated to fostering HIV resilience. Researchers, ASOs, and other community-based agencies may be able to find novel ways to incorporate the value of compartmentalizing as a behavioral strategy in the conceptualization and development of their interventions to foster resilience to HIV/AIDS among gbMSM. For example, they may want to consider evaluating and developing interventions that would provide safe spaces for gbMSM living with HIV/AIDS who are at stages in their lives when compartmentalizing is still the most convenient and viable option for them to cope, while at the same time offering them alternative interventions to consider that could lead to more definitive solutions to their challenges. This two-tier approach to simultaneously providing safe spaces for compartmentalizing while prospectively offering other available interventions has the potential to address health disparities that impact gbMSM living with HIV/AIDS who are at a complex crossroads in their lives.

*Serosorting* is the practice of preferentially having sex with partners that have a concordant HIV status or selectively using condoms with HIV-discordant partners [41]. In the narratives of our participants, serosorting primarily meant choosing to have sex only with other HIV-positive gbMSM. Serosorting is a common practice among both HIV-positive and HIV-negative gbMSM but is usually more commonly practiced by men who have recently been diagnosed with HIV. The gbMSM substantially modify their sexual behavior after being diagnosed with HIV, and sustain this behavior for several years, which is a practice that some researchers assert may reduce HIV transmission to HIV-negative gbMSM [42]. Research showed that decisions to serosort for HIV risk reduction were traditionally based on personal impressions and beliefs and that there is limited guidance on serosorting offered to the gbMSM community from public health services, including services that are offered by ASOs and other community-based agencies [43]. This lack of guidance on the practice of serosorting is evident in the available interventions of many ASOs for HIV prevention and is in all likelihood due to conflicting or precipitously changing information on the effectiveness of serosorting as a behavioral protective factor and strategy. Case in point, early reports concluded that serosorting offers only limited protection from HIV/AIDS [41], while other reports have determined that serosorting is associated with a modest decrease in risk of HIV infection [44]. Still, other reports stated that serosorting’s protective effect is not statistically significant, contending that consistent condom use is still the most protective of all strategies [45]. Most recently, Goodreau and colleagues [46] noted two meta-analyses, which found that men practicing serosorting had lower HIV incidence than those who practiced condomless anal sex without regard to partner status. Some researchers were fervent to point out that pre-exposure prophylaxis and other biomedical prevention strategies (e.g., post-exposure prophylaxis, treatment as prevention) may have intertwined with, rather than supplanted, serosorting and other seroadaptive behaviors (i.e., selecting specific sexual acts (i.e., oral vs. anal), considering sexual positioning (i.e., insertive vs. receptive), and making decisions surrounding condom use), as well as changed serosorting patterns in the last decade [46,47]. These changes in seroadaptive behaviors and serosorting patterns, along with more definitive, evidence-based data on the effectiveness of serosorting as a behavioral strategy, could prove to be crucial aspects to consider in the development of future interventions that are meant to provide reliable guidance on serosorting, foster HIV resilience among gbMSM, and address health disparities that continue to impact them.

Much like the existing academic literature on serosorting and compartmentalizing related to sexual activities and HIV/AIDS, scholarly discourse on *volunteering* among HIV-positive gbMSM has essentially not focused on the value of volunteering as a factor that would foster resilience to HIV/AIDS or address health disparities that affect sexual minority men. Empirical research on gbMSM living with HIV/AIDS volunteering their time and energy to support ASOs predominantly revolved around investigations of the benefits of and barriers to the meaningful and greater involvement of people living with HIV/AIDS (MIPA/GIPA) in the development and maintenance of HIV services that are important to them, program and policy development, and the emergence of rights-based and community development approaches in population health [48,49,50,51]. However, unlike serosorting and compartmentalizing as factors that offer protective effects against the clinical and social impacts of HIV/AIDS, volunteering has long been established as a factor that has been consistently considered and valued as an indispensable resource in the development and rollout of interventions, services, programs, and policies in ASOs [49,50,51]. The volunteer work of gbMSM led the way in developing community-based support for people living with HIV/AIDS. Based on our findings, ASOs and other community-based organizations that rely on volunteers for manpower should also recognize the substantial opportunities for volunteers to consequently gain protective effects against the clinical and social impacts of HIV/AIDS, foster HIV resilience, and help address HIV/AIDS health disparities that disproportionately affect them. With this recognition comes additional greater purposes for offering volunteer opportunities in their organizations, and the options to maximize the benefits of volunteering for gbMSM living with HIV/AIDS by intentionally customizing their assigned volunteer work to work that is even more meaningful and resilience fostering, such as work that provides them opportunities to help other gbMSM navigate the landscape of aging with HIV/AIDS [50].

### 4.3. Considering and Valuing Controversial Protective Factors in the Development of Interventions That Foster Resilience to HIV/AIDS

In their own unique ways, the remaining four factors that we identified from the analysis of our interview data (i.e., *abstinence*, *trauma from the loss of many lives to HIV/AIDS at the start of the epidemic*, “*managed*” *substance use*, and *meaningful sexual relationships*) sparked controversy in the academic literature on HIV/AIDS. In fact, because of their seemingly controversial nature, these factors were discussed extensively in empirical research articles [52,53,54,55,56,57,58,59,60], which could potentially be used as starting points when considering these factors in the development of interventions for addressing HIV/AIDS health disparities that affect older gbMSM. We contend that these factors, despite the controversies they may have inspired in prior scholarly contributions, should be seriously considered, and even valued, in the development of interventions for fostering resilience to HIV/AIDS and addressing health disparities, principally because they were purposefully harnessed from the insights and personal experiences of racially and ethnically diverse, older gbMSM living with HIV/AIDS who have exhibited resilience since their HIV diagnosis. In terms of the potential value of each factor that can be utilized in the development of interventions to foster HIV resilience among gbMSM and address HIV/AIDS health disparities that disproportionately affect them, we maintain that there are important considerations to review and assess.

The main controversy that surrounds incorporating *abstinence* in the development and establishment of interventions addressing HIV/AIDS health disparities that impact gbMSM is the debate on whether it would be more effective to promote abstinence-only measures or advocate for abstinence-plus measures (i.e., abstinence plus fidelity and condom use) programs instead [54,61,62,63,64]. With this ongoing debate, many ASOs and community-based agencies find themselves having to select one option over the other in the development of their interventions to address HIV/AIDS health disparities. Further empirical research on this issue may help to clarify which option would be a better choice.

When considering the *trauma from the loss of so many lives to HIV/AIDS at the start of the epidemic* as a factor that may prove useful in the development of interventions to address HIV/AIDS health disparities unduly affecting gbMSM, the question that begs to be asked is: How can ASOs and advocacy groups seeking to foster resilience to HIV/AIDS among gbMSM utilize the lessons learned from such emotionally disturbing loss? During the first decade of the HIV/AIDS epidemic, gbMSM began to experience multiple and unrelenting losses, with many facing existential reflections about death and their own mortality [65]. The loss of loved ones and significant others to HIV/AIDS was experienced by friends, family, caregivers, and partners, and scholars began to view this harrowing loss as a variant of complicated bereavement and complex grief [65,66], then later, as HIV-related post-traumatic stress disorder [59]. Subsequently, some researchers proposed the prospect of considering the importance of this bereavement and grief in the development of resilience interventions [60], with several researchers optimistically investigating the potential benefits that may result from what they have interpreted or deemed as post-traumatic growth [67,68,69]. Although it would be impractical, unethical, and absurd to suggest that trauma from the tragic loss of lives to HIV/AIDS be replicated, or even simulated, in order to generate similar benefits from post-traumatic growth, it would be interesting to see whether and how health experts and social scientists could find innovative ways to develop interventions for addressing HIV/AIDS health disparities utilizing the knowledge that was gained from the examination of the trauma gbMSM have experienced from the catastrophic loss of so many lives to HIV/AIDS. Experts and scientists would need to find creative ways to develop interventions utilizing such knowledge without the use of strategies based on different types of fear that are associated with HIV/AIDS since the response to these fear-based strategies was not always positive [60,66,67,69].

Older adults living with HIV/AIDS have higher rates of substance use than their HIV-negative peers, making it more challenging for ASOs and concerned advocacy groups to address their healthcare needs [70]. Researchers documented that problematic substance use undercuts the success of HIV treatments and secondary prevention efforts, which is a relevant consideration for designing adjuvant interventions for HIV care and prevention [71,72]. Some participants in our study asserted that with *“managed” substance use*, they were able to get through the darkest days of their lives after being diagnosed with HIV. Their assertion raises the concern as to whether “managed” substance use could actually be a factor that needs to be considered or valued in the development of interventions for addressing health disparities that affect gbMSM living with HIV/AIDS. Pragmatically, the most productive way to consider the prospective value of “managed” substance use in fostering resilience to HIV/AIDS is to perhaps follow or support the philosophy and rationale behind harm reduction programs [73,74]. ASOs and other community-based agencies have supported harm reduction programs for many years [75,76], and this route would likely be the most ideal way to consider forging ahead, until such time as more empirical research can provide new evidence on how to best support gbMSM living with HIV/AIDS who feel that their only available recourse to cope with challenges at a particular period in their life would be to rely on “managed” substance use.

In the presentation of our findings, we used the phrase “*meaningful sexual relationships*” to operationalize the notion that many of our participants had diverse types of relationships with other gbMSM over the years that started as sexual, but over time, they became meaningful in terms of other relevant aspects. Participants revealed that these meaningful relationships have provided them protective effects against the clinical and social impacts of HIV/AIDS, and have been essential to fostering their HIV resilience as they age. Abundant recent research was conducted on the social and intimate contexts of these diverse types of gbMSM sexual relationships, the relationship dynamics immersed in them, and the value of possessing the necessary behavioral and communication skills to negotiate sexual agreements in such sexual relationships [55,57,77,78,79,80,81]. This recent body of research is of critical importance to informing, influencing, and impacting the HIV care and prevention interventions that ASOs and other community-based agencies are intending to develop to address health disparities that affect gbMSM, particularly those with intersecting, marginalized identities. According to this body of research, as many as two-thirds of new HIV infections among gbMSM in North America were attributed to primary relationships in which condomless anal sex is more common than it is in casual relationships [55,77,78]. These primary relationships, which involve two or more people who have significant commitments that involve merging the daily infrastructure of their lives in a spouse-like arrangement without specific regard to sexual monogamy and/or emotional connection, have recently garnered greater attention from HIV researchers. This greater attention became apparent as an increasing number of HIV prevention research efforts that were designed to study and intervene on HIV risk within gbMSM partnerships turned their focus to the relevance of these primary relationships [82]. There is much work and empirical investigation that still needs to be done so that future interventions to foster HIV resilience and address HIV/AIDS health disparities could have sufficient evidence-based knowledge on meaningful sexual relationships of gbMSM living with HIV/AIDS to utilize, beginning with the essential aspects within the primary and other meaningful relationships our participants identified and described that contributed to fostering their own resilience to HIV/AIDS.

### 4.4. Recognizing the Different Ways That Protective Factors Foster Resilience to HIV/AIDS

It is important to note that the protective factors we identified during our study helped our participants foster their resilience to HIV/AIDS in different ways, particularly when considering them as factors in the development of interventions for addressing HIV/AIDS health disparities. For example, factors such as *education*, *social support from family and friends*, *volunteering*, and *meaningful sexual relationships* were all associated with the improved use of important health services in the community and positive personal clinical health outcomes among our participants. Factors such as *religion and spirituality*, *compartmentalizing*, and *“managed” substance use*, on the other hand, were factors that helped our participants to cope with the social impacts of HIV/AIDS by buffering the adverse effects that stigma and discrimination brought to their lives. Finally, inasmuch as factors such as *serosorting*, *abstinence,* and recognizing *the traumatic loss of many lives to HIV/AIDS at the start of the epidemic* are known to be factors that are linked with efforts for preventing HIV transmission between gbMSM, these factors also consequently helped to foster the HIV resilience of our participants because they gave our participants the gratifying sense of having the capacity, options, and opportunities to support HIV prevention interventions and concurrently contribute to the goal of ending the HIV/AIDS epidemic (a goal that is very meaningful to them as gbMSM living with HIV/AIDS), and thus, foster their resilience to HIV/AIDS.

## 5. Conclusions

In developing interventions that are dedicated to addressing HIV/AIDS health disparities that disproportionately affect gbMSM, it would be important for ASOs, LGBTQ+ agencies, and community-based advocacy groups to consider and value factors that our participants identified as protective against the clinical and social impacts of HIV/AIDS. The unique contribution of our study to the current HIV resilience and health disparities academic literature lies in the knowledge that was purposefully derived from the insights and lived experiences of racially and ethnically diverse, older gbMSM living with HIV/AIDS on protective factors that have fostered their HIV resilience over the years. Based on their insights and personal experiences in fostering their own resilience to HIV/AIDS, our participants identified a few protective factors that have not only already been established in prior academic literature as resilience fostering but have also been incorporated by ASOs, program planners and evaluators, and advocates in successful HIV care and prevention interventions over the years. More importantly, our participants also identified numerous behavioral and controversial protective factors, which were not specifically discussed in prior research in the context of fostering the resilience of gbMSM to HIV/AIDS. Some of these behavioral and controversial protective factors may have already been considered in the development of interventions outside the context of resilience fostering, while the other factors, as we have determined, have yet to be considered, further explored, or extensively examined through future empirical studies in order to reveal and document their full value. Numerous factors that currently are or would likely prove worthwhile to consider in the development of HIV care and prevention interventions for fostering resilience to HIV/AIDS and addressing HIV/AIDS health disparities affecting gbMSM were presented in this paper, and further research is needed to keep the momentum of the ongoing development of these interventions moving forward.

## Figures and Tables

**Table 1:** Participant characteristics (*n* = 41).

**Race/Ethnicity**	***n*** **(%)**
Aboriginal	2 (5)
African/Caribbean/Black	8 (20)
Hispanic/Latino	4 (10)
White	17 (41)
South/Southeast Asian	7 (17)
West Asian/Middle Eastern	3 (7)
**Age Range**	***n*** **(%)**
40–44 years old	7 (17)
45–49 years old	8 (20)
50–54 years old	10 (24)
55–59 years old	7 (17)
60–64 years old	5 (12)
65–69 years old	1 (3)
> 70 years old	3 (7)
**Identified as**	***n*** **(%)**
Gay	30 (73)
Bisexual	7 (17)
Two-spirit	3 (7)
MSM	1 (3)
**Geographical Location**	***n*** **(%)**
Downtown Toronto	30 (73)
Greater Toronto Area	8 (20)
Southwestern Ontario	3 (7)
